# Carotid body paraganglioma

**DOI:** 10.1002/ccr3.2337

**Published:** 2019-07-30

**Authors:** Suwanna Pitchaiprasert, Patchaya Boonchaya‐Anant, Thiti Snabboon

**Affiliations:** ^1^ Department of Medicine Surin Hospital Surin Province Thailand; ^2^ Division of Endocrinology and Metabolism, Department of Medicine, Faculty of Medicine Chulalongkorn University Bangkok Thailand; ^3^ Excellence Center in Diabetes, Hormone and Metabolism King Chulalongkorn Memorial Hospital, Thai Red Cross Society Bangkok Thailand

**Keywords:** carotid body, flow‐void sign, paraganglioma, salt‐pepper appearance

## Abstract

Carotid body paraganglioma is commonly asymptomatic, slow‐growing, and nonfunctioning. With its relative contraindication to biopsy due to its high vascularity in nature, imaging characteristics are the key to help making the diagnosis. Treatment modalities, ranging from an observation to definitive treatment with surgery, should be selected on an individual basis.

## 1 CASE

**Figure 1:**
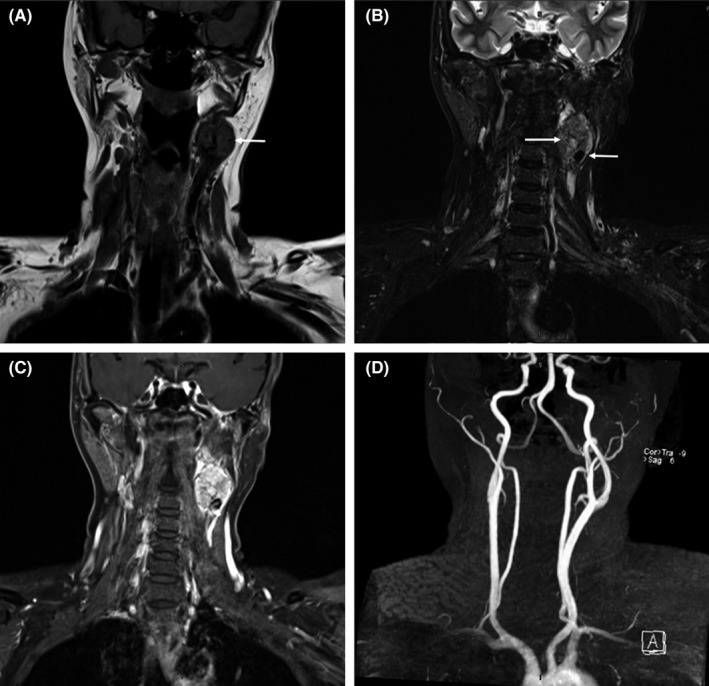
MRI of the neck, coronal view showed a heterogeneous mass with flow‐void sign and salt‐and‐pepper appearance (arrow) at left carotid bifurcation, characterized by iso‐signal intensity on T1‐weighted image A, hypersignal intensity on T2‐weighted image B, and intense gadolinium enhancement C. MRA demonstrated a prominent size of left ascending pharyngeal artery which provided feeding vessels into the mass D

A 53‐year‐old woman presented with a painless pulsatile left neck mass. MRI study revealed a 2.7 × 3.1 × 3.9 cm well‐circumscribed hypervascular mass at left carotid bifurcation. The mass was isointense in T1W (Figure 1A), heterogeneous hyperintense in T2W (Figure 1B), and intensely enhancing after gadolinium injection (Figure 1C). Flow‐void sign with salt‐and‐pepper appearance was also noted (arrow). MR angiography demonstrated a tumor splaying at the carotid bifurcation (Figure 1D). The findings from the MRI support the diagnosis of a paraganglioma.

## 2 DISCUSSION

Carotid body paraganglioma, the most common type of head and neck paraganglioma, is located at the bifurcation of the common carotid artery. It is commonly asymptomatic, sporadic, and benign. The tumor is typically mobile in the horizontal plane and associated with bruits or thrills. With the relative contraindication to biopsy due to its high vascularity in nature, the MRI features play a pivotal role in the diagnosis. In addition, the mass effect is typically seen in splaying the internal and external carotid arteries (lyre sign). Flow‐void sign is a feature of the hypervascularity of the lesion, which results in multiple signal drop (“pepper”) areas interspersed with hyperintense (“salt”) foci.^1^ Nuclear imaging techniques including somatostatin receptor scintigraphy or positron emission tomography scans may be used to evaluate for multicentric or metastatic diseases.^2^ Treatment ranges from an observation to a combination of multiple modalities including surgery, embolization, or conventional/stereotactic radiotherapy. In our case, conventional radiotherapy was considered a more appropriate treatment option.

## CONFLICT OF INTEREST

None declared.

## AUTHOR CONTRIBUTIONS

SP and TS: conceptualized and designed the study, drafted the initial manuscript; PB and TS: revised the manuscript; SP, PB, and TS: critically reviewed the manuscript for important intellectual content and approved the final version.
